# α-Glucosidase Inhibitory Activity of Fermented Okara Broth Started with the Strain *Bacillus amyloliquefaciens* SY07

**DOI:** 10.3390/molecules27031127

**Published:** 2022-02-08

**Authors:** Yifang Gao, Wenhui Bian, Yuanyuan Fang, Peng Du, Xueting Liu, Xueru Zhao, Fengjuan Li

**Affiliations:** Key Laboratory of Food Nutrition and Safety, Tianjin University of Science and Technology, Ministry of Education, No. 29 13th Avenue, Teda, Tianjin 300457, China; 15147652800@163.com (Y.G.); bianwenhui0717@163.com (W.B.); zlian1983@163.com (Y.F.); yangwenqing00000@163.com (P.D.); liuxuetingapple@163.com (X.L.); 15227439583@163.com (X.Z.)

**Keywords:** *Bacillus amyloliquefaciens* SY07, α-glucosidase inhibitory activity, 1-deoxynojirimycin (DNJ), okara, inhibition kinetics

## Abstract

In this work, a new strain of *Bacillus amyloliquefaciens* SY07 isolated from a traditional fermented soybean food was reported to possess remarkable α-glucosidase inhibitor-producing ability. Different culture media were applied for the proliferation of *B.* amyloliquefaciens SY07, and it was found that fermented okara broth presented the highest α-glucosidase inhibitory activity, while Luria-Bertani medium showed a negative effect. The extract from fermented okara broth acted in a dose-dependent manner to inhibit α-glucosidase activity, with an IC_50_ value of 0.454 mg/mL, and main inhibitors in the fermentation extract presented a reversible, uncompetitive pattern according to Lineweaver–Burk plots. Moreover, 1-deoxynojirimycin, a recognized α-glucosidase inhibitor, was found in the extract. Results indicated that *B. amyloliquefaciens* SY07 could utilize okara, a by-product from the soy processing industry, to generate α-glucosidase inhibitors effectively, and be regarded as a novel excellent microbial candidate for safe, economical production of potential functional foods or ingredients with hypoglycemic effect.

## 1. Introduction

Diabetes mellitus, a metabolic disorder characterized by chronic hyperglycemia and accompanied by a number of severe complications, is a worldwide epidemic. α-Glucosidase (EC 3.2.1.20), present in the small intestinal brush border, can catalyze the transformation of complex carbohydrates into glucose suitable for absorption [1]. The inhibition of α-glucosidase has been well established as an effective approach to the management of non-insulin-dependent diabetes, suppressing oligosaccharide hydrolysis and thus reducing postprandial carbohydrate uptake. Some synthetic α-glucosidase inhibitors, such as acarbose and voglibose, are widely used as hypoglycemic agents in clinics, but they also cause various undesirable side-effects including flatulence, nausea, and diarrhea [2,3]. Therefore, the search for safer natural inhibitors has drawn considerable attention.

Recently, many α-glucosidase inhibitory components were reported from plant or foodstuff sources such as morning glory [4], flower buds of *Lonicera japonica* [5], Graviola leaf [6], *Hibiscus sabdariffa* [7], *Salacia oblonga* [8], grape pomace [9], and so forth. Comparatively, fast-proliferating microorganisms present specific characteristics, in particular producing bioactive metabolites in a cost-effective way. Certain species of *Streptomyces* [10], *Actinoplanes* [11], *Flavobacterium* [12] have been reported to have the capacity of generating α-glucosidase inhibitors, which underlay the development of currently commercially available antidiabetic drugs. Recently, some novel bacterial strains were also discovered to be capable of producing α-glucosidase inhibitors, such *Bacillus subtilis* B2 [13], *Bacillus amyloliquefaciens* AS385 [14], *Bacillus methylotrophicus* K26 [15], and *Paenibacillus* sp. TKU042 [16]. These studies suggest the promising application of *Bacillus* as potential excellent microorganisms in the treatment of hyperglycemia.

The metabolic activity of microorganisms, including possible production of α-glucosidase inhibitors, might be influenced by different fermentation conditions. Previously, we isolated a strain known as *Bacillus amyloliquefaciens* SY07 with a remarkable ability to produce α-glucosidase inhibitors, and its fermentation conditions—such as the inoculation amount, initial pH and temperature—were optimized [17]. In this sense, in the present work, the inhibition of α-glucosidase activity by different culture broths started with *B. amyloliquefaciens* SY07 was further investigated. The inhibition kinetics of the fermented broth extract with potent anti-α-glucosidase activity as well as the bioactive inhibitory compound were also analyzed.

## 2. Results and Discussion

**Figure 1:**
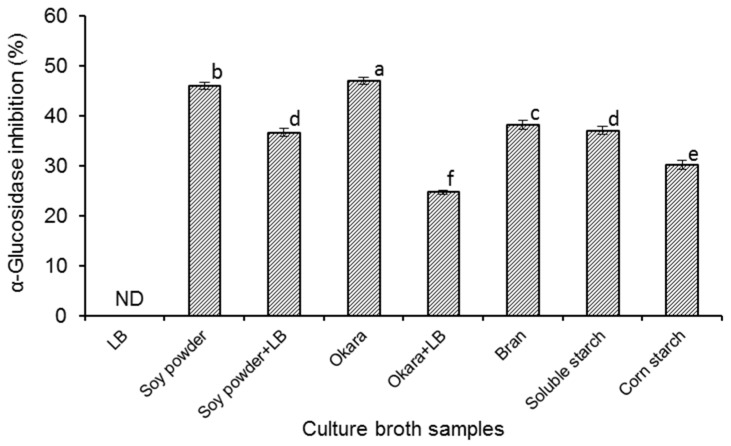
The α-glucosidase inhibitory activities of different culture broths started with *B. amyloliquefaciens* SY07. Results are expressed as percentage α-glucosidase inhibition by the filtrated culture broth solution further diluted 5 fold. ND indicates no α-glucosidase inhibitory activity detected. Data are the means of triplicate analyses, with different letters indicating significant difference at *p* < 0.05.

The α-glucosidase inhibitory activities of different culture broths started with *B. amyloliquefaciens* SY07 were evaluated (Figure 1), and it was found that all the fermented samples (except the sample prepared in LB medium) showed α-glucosidase inhibitory activities with percentage inhibition ranging from 24.72 to 47.07% under the experimental conditions. It has been reported that α-glucosidase inhibitors can be found from the metabolites of certain microorganisms, such as *Streptomyces* [10], *Actinoplanes* [11], *Flavobacterium* [12] as well as some lactic acid bacteria [18]. In particular, after the report of a strain of *B. subtilis* B2 by Zhu et al. [19], Onose and co-workers [14] reported a strain of *B. amyloliquefaciens* AS385 with great mass production of 1-deoxynojirimycin. In several previous studies, it was found that some strains of *B. amyloliquefaciens* could exert fibrinolytic activity [20] and antibacterial activity [21]. However, there is still quite limited information on *B. amyloliquefaciens* species with α-glucosidase inhibitor-producing ability. In the present work, we gained a new strain of *B. amyloliquefaciens* SY07, and the good α-glucosidase inhibitory activity of its culture broth could make it a novel potential microbial source of antidiabetic products.

As shown in Figure 1, different *B. amyloliquefaciens* SY07-fermented broths showed varying α-glucosidase inhibitory activities, providing evidence for the important role of medium components in inducing the generation of α-glucosidase inhibitors by *B. amyloliquefaciens* SY07. No α-glucosidase inhibitory activity was observed in the fermentation broth of LB medium. When *B. amyloliquefaciens* SY07 was cultured in LB medium supplemented with soy powder or okara, the α-glucosidase inhibitory activities of the broths were also significantly (*p* < 0.05) weaker than that fermented in sole soy powder or okara culture, respectively. In the study of Zhu et al. [19], *B. subtilis* B2 could produce α-glucosidase inhibitors in LB medium, though it was less than that in the medium supplemented with soy powder, okara, or starch. LB medium is normally recognized as a suitable culture for the proliferation of *Bacillus*. However, in this work, it was found that LB medium exerted negative effect on starting the synthesis of α-glucosidase inhibitors by *B. amyloliquefaciens* SY07. Onose et al. [14] reported that sorbitol supplementation in the growth medium could enormously elevate the yield of 1-deoxynojirimycin by *B. amyloliquefaciens* AS385 via increasing mRNA expression of certain biosynthetic gene. Therefore, it is of great significance to further investigate the production mechanism of α-glucosidase inhibitors by *B. amyloliquefaciens* SY07. On the other hand, from Figure 1, the highest α-glucosidase inhibitory activity (47.07%) was detected in fermented okara broth. Since okara is the main by-product of the soybean processing industry, it would present noticeable economical merit while using okara as culture medium of *B. amyloliquefaciens* SY07 to obtain potential hypoglycemic food products.

**Figure 2:**
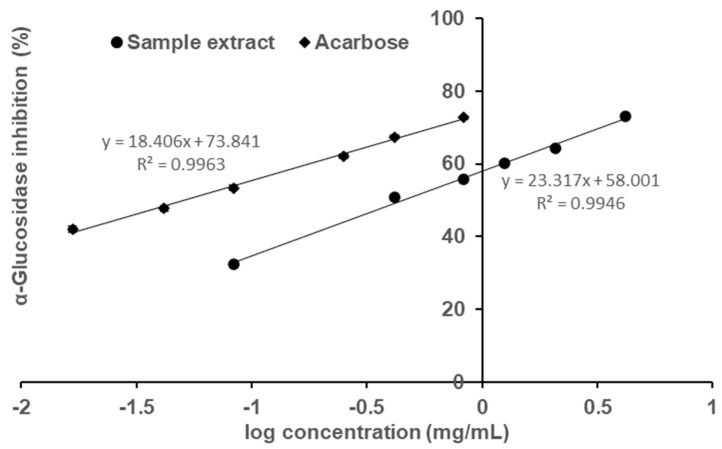
Profiles of concentration−dependent inhibition of α−glucosidase activity and IC_50_ value of fermented okara broth extract, with acarbose serving as control. The IC_50_ values for the sample extract and acarbose on α−glucosidase inhibition are 0.454 and 0.051 mg/mL, respectively. Data are the means of triplicate analyses.

**Figure 3:**
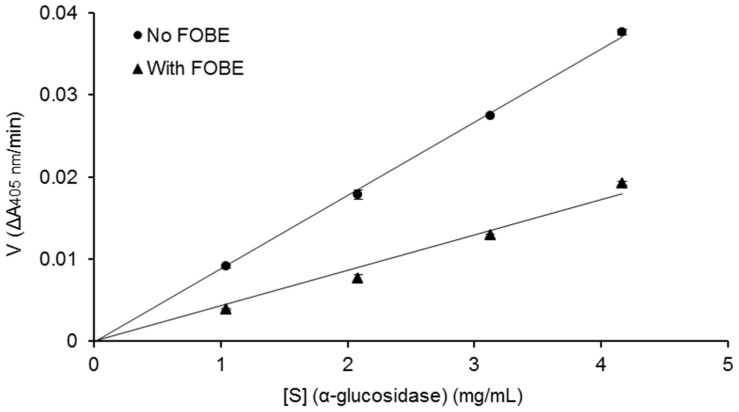
Relationship between enzymatic reaction velocity and enzyme concentration in the absence (▲) and presence (0.667 mg/mL) (●) of fermented okara broth extract (FOBE). Data are the means of triplicate analyses.

**Figure 4:**
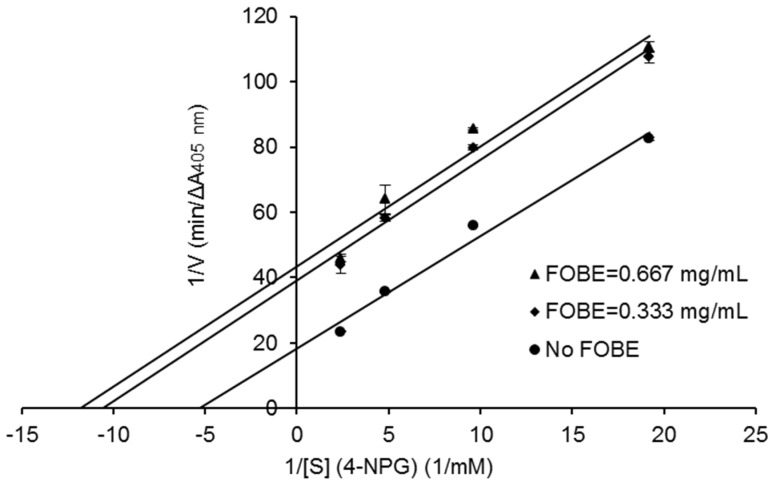
Lineweaver−Burk plots of the inhibition on α−glucosidase activity by fermented okara broth extract (FOBE). α−Glucosidase inhibitory activity was measured in the absence (●) and presence of 0.333 (♦) or 0.667 mg/mL (▲) of the sample extract. X axis represents the reciprocal of the substrate concentration ([S]) and Y axis represents the reciprocal of the reaction rate (V) indicated by absorbance intensity unit per minute. Data are the means of triplicate analyses.

The sample, fermented okara broth extract (FOBE), which possessed the strongest α-glucosidase inhibitory activity was subjected to further analysis of inhibition properties. As depicted in Figure 2, the profiles of percentage inhibition versus sample concentration showed that FOBE inhibited α-glucosidase activity in a dose-dependent manner, with an IC_50_ value of 0.454 mg/mL. The positive control acarbose, a clinically used hypoglycemic drug, presented an IC_50_ value of 0.051 mg/mL. Compared to the literature, the α-glucosidase inhibitory activity of FOBE was much higher than some plant extracts reported, such as the crude fraction from Graviola Leaf (IC_50_ = 1.38 mg/mL) [6], *Hibiscus sabdariffa* (IC_50_ = 6.00 mg/mL) [7], and *Salacia oblonga* (IC_50_ = 80.90 mg/mL) [8]. Moreover, from Figure 3, the relationship between enzymatic reaction velocity and enzyme concentration showed that FOBE exhibited a reversible inhibition against α-glucosidase activity. As shown in Figure 4, the Lineweaver–Burk plots further indicated that main α-glucosidase inhibitors in FOBE were inclined to act in an uncompetitive pattern, with a concentration-dependent decrease in both Vmax and Km values. Results suggested that the inhibitors tended to be stable and easily combined with the α-glucosidase-substrate complex, and caused a loss of α-glucosidase activity by affecting the conformation of its enzymatic active site.

**Figure 5:**
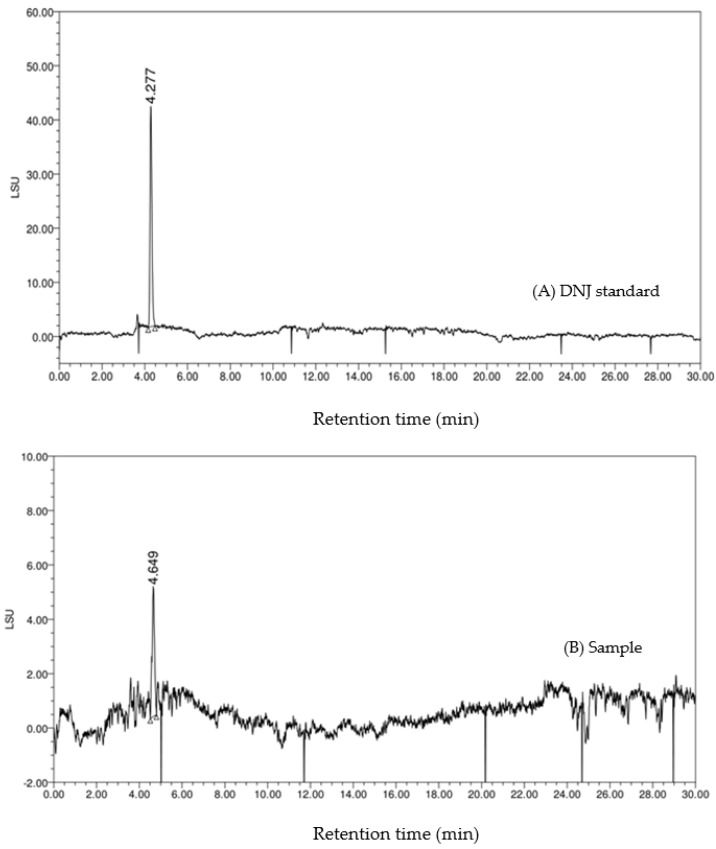
HPLC chromatograph with ELSD detection. (**A**) HPLC chromatogram of the standard DNJ; (**B**) HPLC chromatogram of the sample.

**Figure 6:**
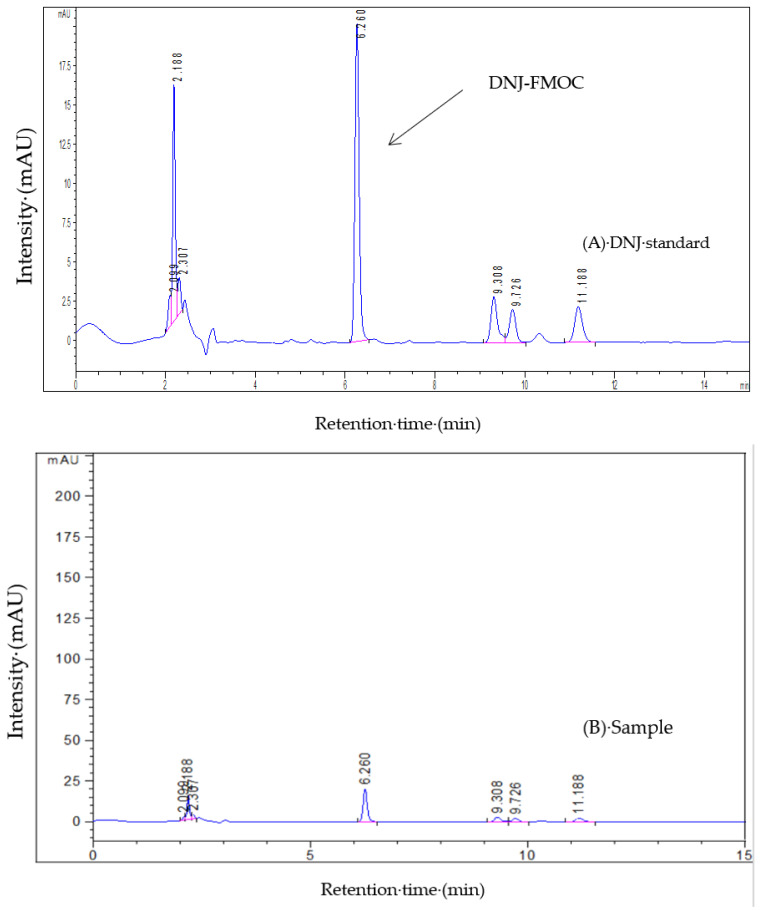
HPLC chromatograph with VWD detection. (**A**) HPLC chromatogram of the standard DNJ; (**B**) HPLC chromatogram of the sample.

**Figure 7:**
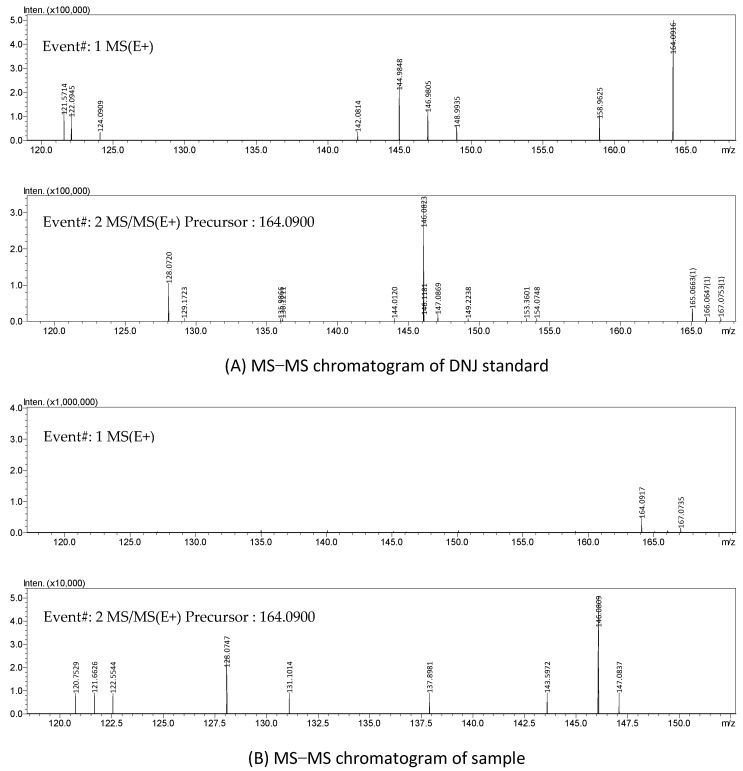
Full scan MS–MS spectrum of the standard DNJ and the sample.

Moreover, the HPLC-ELSD chromatography of the standard DNJ and the sample is shown in Figure 5. On the chromatogram (B), the retention time of the main peak was 4.649 min, which was similar to that of the DNJ standard (4.277 min). The HPLC-VWD chromatography is shown in Figure 6. The FMOC-derivatized DNJ (retention time: 6.260 min) was completely separated on the chromatogram (A), while, similarly, on the chromatogram (B) of the sample, there was an obvious peak at 6.260 min, which suggested the presence of DNJ in FOBE. After dialysis, in the full-scan MS spectrum, a peak at *m*/*z* 164.09 (M^+^+1) is shown in Figure 7. The fragment ions (main daughter ions *m*/*z* 128.07 and 146.08) of the precursor (*m*/*z* 164.09) were shown in MS 2. Compared to the MS–MS spectrum of the standard DNJ, the fragment ion information of the MS 2 of the sample was almost the same as the standard DNJ, which further indicated that the generation of DNJ in the fermented okara broth started with *B. amyloliquefaciens* SY07. In addition, the strain processed okara with heterofermentation, and apart from DNJ, there were other bioactive substances produced. Therefore, it would be of great significance to further investigate more α-glucosidase inhibitors as well as their metabolic pathways in future studies.

## 3. Materials and Methods

### 3.1. Microorganism and Materials

*B. amyloliquefaciens* SY07 was isolated from a traditional fermented soybean food and identified based on 16S rRNA gene and gyrB gene sequences as well as morphological, physiological, and biochemical properties by the Institute of Microbiology, Chinese Academy of Sciences (Beijing, China). α-Glucosidase (from rat intestinal acetone powder), 4-nitrophenyl-α-d-glycopyranoside (4-NPG), cornstarch, and soluble starch were purchased from Sigma-Aldrich, Inc. (St. Louis, MO, USA). Acarbose was purchased from Bayer Schering Pharma (Leverkusen, Germany). Soybeans were purchased from the Center of Soybean Research, Agricultural Academy of Jilin Province (Jilin, China), and ground into powder prior to use. Okara and wheat bran were kindly provided by Tianjin Food Processing and Engineering Research Center (Tianjin, China). All other reagents were of analytical grade.

### 3.2. Preparation of Starter Culture

*B. amyloliquefaciens* SY07 was inoculated into Luria-Bertani (LB) liquid medium (Oxoid, UK) and incubated at 37 °C for 12 h in an incubation shaker. The enriched culture was diluted with sterile distilled water to prepare the starter suspension with a concentration of around 10^7^ cfu/mL. 

### 3.3. Production of α-Glucosidase Inhibitors in Different Growth Media

Different growth media were used to culture the bacteria, and LB medium was employed as control. Soybean powder (5%), okara (5%), cornstarch (5%), soluble starch (5%), or wheat bran (5%), respectively, was suspended in water to form five kinds of culture media. Two other kinds of media were prepared by adding soybean powder (5%) or okara (5%) into LB medium, respectively. The initial pH of the medium was regulated to 7.0. An amount of 60 mL of the growth medium was placed in flasks (250 mL) and sterilized at 121°C for 20 min in an autoclave. Then, each medium was inoculated with the starter culture (2%, *v*/*v*) and incubated at 37 °C for 48 h in the incubation shaker.

### 3.4. Preparation of Sample Extracts

After fermentation, the culture broth was heated in a boiling water bath for 15 min, cooled, and then centrifuged (Avanti J-26 XP, Beckman Coulter Inc., Brea, CA, USA) at 8000 rpm for 25 min at room temperature. Part of the resulted supernatant was collected after filtration by a 0.45 μm membrane and then used for α-glucosidase inhibitory activity assay. For further analysis of inhibition kinetics and inhibitor identification, the remaining supernatant obtained by centrifugation was collected after filtration through 125 mm filter paper (Advantec, Tokyo, Japan), followed by vacuum evaporation at 60 °C and then lyophilization with a freeze dryer (ALPHA 2-4 LD plus, Marin Christ Co., Osterode, Germany). The dried sample was stored at −20 °C prior to use.

### 3.5. Assay for α-Glucosidase Inhibitory Activity

α-Glucosidase inhibitory activity was determined according to the method previously reported [22], by measuring the release of 4-nitrophenol from the substrate 4-NPG which could be hydrolyzed by α-glucosidase. Briefly, the sample solution (20 μL) was mixed sufficiently with 120 μL of 0.5 M phosphate buffer (pH 6.7) and 50 μL of 4-NPG (0.9133 mg/mL). After adding 50 µL of α-glucosidase solution (25 mg/mL), the reaction mixture was allowed to stand at 37 °C for 50 min. Then, the reaction was terminated by adding 50 μL of 0.67 M Na_2_CO_3_ solution. The absorbance of the mixture was detected at 405 nm on a microplate reader (Infinite M200 PRO, Tecan Group Ltd., Männedorf, Switzerland). The percentage α-glucosidase inhibition was calculated as follows:

α-glucosidase inhibition (%) = (1 − A_sample_/A_control_) × 100 (1)

where A_sample_ is the absorbance of sample reaction solution in the presence of α-glucosidase inhibitors and A_control_ is the absorbance of control without α-glucosidase inhibition. Dose-dependent inhibition of α-glucosidase activity was evaluated with six different concentrations of the inhibitor. The final concentrations of the fermented okara broth extract were 4.17, 2.08, 1.25, 0.83, 0.42, and 0.08 mg/mL, while the final concentrations of acarbose were 0.83, 0.42, 0.25, 0.08, 0.04, and 0.02 mg/mL. The IC_50_ value was defined as the concentration of the inhibitor required to inhibit 50% of α-glucosidase activity, calculated via the linear function of percentage α-glucosidase inhibition versus logarithm of the inhibitor concentration. Acarbose was served as positive control.

To explore whether the inhibition on α-glucosidase by the potent fermentation sample was reversible, enzymatic reaction velocity was monitored at varying enzyme concentrations, in the absence and presence of the inhibitory sample. Furthermore, the inhibition mode of the sample on α-glucosidase was determined at varying substrate concentrations, in the absence and presence of the inhibitory sample, using Lineweaver–Burk plots.

### 3.6. Separation and Purification

The α-Glucosidase inhibitors were separated with the method previously reported [23], with some modifications. The lyophilized powder of the fermented okara broth was suspended in 80% ethanol and placed at 4 °C for 12 h. After centrifugation at 3000 rpm for 15 min, the supernatant was applied to an Amberlite IR-120 Na^+^ form column, and 1.0 M NH_4_OH elution was collected. The pass-through fraction was concentrated by vacuum evaporation followed by the lyophilization with a freeze dryer.

### 3.7. High-Performance Liquid Chromatography (HPLC) and Mass-Mass Spectrometer (MS-MS) Analysis

The fraction with high inhibitory activity was subjected to HPLC and MS-MS analysis. A TSK-Gel-amide-80 column (4.6 mm × 250 mm, 4 μm, Tosoh, Tokyo, Japan) was used in the HPLC-ELSD system (Shimadzu, Japan). The separation was performed using a mixture of acetonitrile and distilled water (81:19, *v*/*v*, containing 6.5 mM ammonium acetate, pH 5.5) at 1.0 mL/min for 30 min, with a column temperature of 70°C. The temperature of the drift tube was 80 °C, the nebulizing gas was at a pressure of 2.3 bar, and the gain was set at 1. The inhibitors were dissolved in the mobile phase and an amount of 25 μL was applied. The compound 1-deoxynojirimycin (DNJ) was used as standard.

The HPLC-VWD analysis was performed on a CAPCELL PAK C18 column (4.6 × 250 mm, 5 μm) with a detection wavelength of 254 nm. The analyte was eluted with a mobile phase of acetonitrile and 0.1% aqueous acetic acid (40:60, *v*/*v*) at 1.0 mL/min for 40 min. The column temperature was 25 °C. The sample (40 μL) was applied after derivatization with 9-Fluorenylmethyl chloroformate [24].

MS-MS spectrometry analysis was performed on a Shimadzu CMB-20 mass spectrometer (Shimadzu Co., Kyoto, Japan) equipped with an ESI source and an ion trap mass analyzer. The mass range of *m*/*z* was from 50 to 2000. The sample was dissolved in the acetonitrile–water solution (1:1, *v*/*v*), and 3 μL of the sample solution was directly injected for multiple stage (MS–MS) analyses. In the full scan mode, the mass spectrometer was operated over a range of *m*/*z* 50–500 in the positive mode. The standard DNJ was also analyzed by MS-MS under similar conditions to the sample.

### 3.8. Statistical Analysis

Data are the means of triplicate analyses and expressed as means ± standard errors. Data were analyzed using IBM SPSS Statistics version 19.0 (IBM Co., Armonk, NY, USA). Duncan’s multiple range test was used to determine differences among samples. A probability value of less than 0.05 was considered statistically significant.

## 4. Conclusions

In conclusion, a newly isolated strain *B. amyloliquefaciens* SY07 was found capable of producing α-glucosidase inhibitors in certain culture media. The highest α-glucosidase inhibitory activity was detected in fermented okara broth, and the fermentation extract acted in a dose-dependent manner against α-glucosidase activity, with an IC_50_ value of 0.454 mg/mL. It was also found that main inhibitors in fermented okara broth presented a reversible, uncompetitive pattern, and DNJ was one of the bioactive compounds that contributed to the anti-α-glucosidase activity of the fermented broth. Further work is now in progress to explore other potential α-glucosidase inhibitory metabolites generated by the strain. This study provided a novel excellent microbial resource for safe, economical production of α-glucosidase inhibitors, which could be applied for the development of potential functional foods with antidiabetic effect.

## Data Availability

Not applicable.

## Sample Availability

Not available.
